# *Giardia duodenalis* genetic assemblages and hosts

**DOI:** 10.1051/parasite/2016013

**Published:** 2016-03-16

**Authors:** Martin F. Heyworth

**Affiliations:** 1 Research Service (151), Corporal Michael J. Crescenz Veterans Affairs (VA) Medical Center, University and Woodland Avenues Philadelphia PA 19104 USA; 2 Department of Medicine, Perelman School of Medicine, University of Pennsylvania Philadelphia PA 19104 USA

**Keywords:** Assemblage, Genotype, *Giardia*, *Giardia* infections, Giardiasis

## Abstract

Techniques for sub-classifying morphologically identical *Giardia duodenalis* trophozoites have included comparisons of the electrophoretic mobility of enzymes and of chromosomes, and sequencing of genes encoding β-giardin, triose phosphate isomerase, the small subunit of ribosomal RNA and glutamate dehydrogenase. To date, *G. duodenalis* organisms have been sub-classified into eight genetic assemblages (designated A–H). Genotyping of *G. duodenalis* organisms isolated from various hosts has shown that assemblages A and B infect the largest range of host species, and appear to be the main (or possibly only) *G. duodenalis* assemblages that undeniably infect human subjects. In at least some cases of assemblage A or B infection in wild mammals, there is suggestive evidence that the infection had resulted from environmental contamination by *G. duodenalis* cysts of human origin.

## Abstract (fr)

Les techniques pour sous-classer morphologiquement des trophozoïtes identiques de *Giardia duodenalis* ont inclus des comparaisons de la mobilité électrophorétique des enzymes et des chromosomes et le séquençage des gènes codant pour la β-giardine, la triose-phosphate isomérase, la petite sous-unité ribosomique de l’ARN et la glutamate déshydrogénase. À ce jour, *G. duodenalis* a été sous-classé en 8 assemblages génétiques (désignés par A-H). Le génotypage de *G. duodenalis* isolés à partir de divers hôtes a montré que les assemblages A et B infectent le grand plus grand nombre d’espèces d’hôtes, et semblent être les assemblages principaux (ou peut-être uniques) qui infectent les sujets humains de manière indéniable. Dans au moins certains cas d’infection chez les mammifères sauvages par les assemblages A ou B, des éléments indiquent que l’infection était due à la contamination de l’environnement par des kystes de *G. duodenalis* d’origine humaine.

Morphologically similar or identical *Giardia* organisms, designated *Giardia duodenalis* (synonyms *G. intestinalis* and *G. lamblia*) [54], can infect the intestine of numerous species of mammalian host. *G. duodenalis* is the only *Giardia* species that causes human infection; other currently recognised species in this genus include the following (hosts are mentioned in parentheses): *Giardia muris* (rodents) [15], *G. microti* (voles, muskrats) [57], *G. psittaci* (budgerigars) [11], *G. ardeae* (great blue herons) [12] and *G. agilis* (amphibians) [14].

From the 1980s onwards, increasingly precise methods have been developed to sub-classify morphologically identical *G. duodenalis* organisms. Early work of this type involved examination of the electrophoretic mobility of *G. duodenalis* enzymes [1, 23]. In the mid-1990s, such work delineated two distinct sub-populations of *G. duodenalis*, designated assemblages A and B [32]. Additional evidence for heterogeneity of *G. duodenalis* emerged from study of the electrophoretic mobility of *Giardia* chromosomes [46]. Polymerase Chain Reaction (PCR) amplification of *G. duodenalis* DNA, and restriction fragment length polymorphism (RFLP) analysis and sequencing of the resulting PCR products, added further insight into the heterogeneity of the organism, confirming the existence of assemblages A and B, and – in conjunction with data from enzyme electrophoresis – delineating six additional assemblages (C–H) [5, 26, 27, 34–37].

*Giardia duodenalis* genes (genetic loci) used for genotyping the organisms include genes encoding β-giardin (bg), triose phosphate isomerase (tpi), the small subunit of ribosomal RNA (ssu) and glutamate dehydrogenase (gdh) [15]. *Giardia duodenalis* assemblages have been shown to be either relatively specific to certain hosts (assemblages C–H) or essentially unrestricted in terms of the species of host that they can infect (assemblages A and B; Table 1). Within a single “isolate” of *G. duodenalis*, different genetic loci may have DNA sequences typical of different assemblages (e.g., ssu typical of assemblage B, and tpi and bg typical of assemblage A) [39], a situation that may make it unrealistic to try to assign a given isolate of *G. duodenalis* exclusively to one or other assemblage. This point is pertinent to Table 1, which may present an oversimplified classification, in not discriminating between data obtained from a single genetic locus and from several loci [7]. A comprehensive review, published in 2011, includes detailed information about assemblages of *G. duodenalis*, and non-human hosts for the respective assemblages [15].

**Table 1.** *Giardia duodenalis* assemblages and corresponding hosts.

Assemblage	Host(s)
A	Humans [15], dog [5, 8, 29, 58], cat [27, 51], cattle [15, 24], alpaca [19], deer [45, 48], ferret [39], pig [3], beaver [40], chinchilla [39], jaguar [47], horse [56], marsupials [38, 52], sheep [17, 27, 30, 62], goat [17], muskox [9], non-human primates [60, 61], cetacean(s) [41, 42], seals [26], Australian sea lion [10], moose [27, 43], reindeer [43], chicken [5], gull [26]
B	Humans [5, 15], cattle [15, 38], dog [8], gazelle [48], deer [38], horse [56], beaver [40, 50], muskrat [50], chinchilla [39, 47], ferret [39], rabbit [30, 39], Desmarest’s hutia [39], marsupials [38, 52], guinea pig [27], rock hyrax [4], non-human primates [47, 60, 61], chicken [5], sheep [62], seals [26], pig [13], Australian sea lion [10], ostrich [47], dolphin [25, 41], porpoise [25], gull [26]
C	Dog [8, 29], kangaroo [38], cattle [33], pig [33] cetacean(s) [42]
D	Dog [5, 8], chinchilla [39], kangaroo [38], cattle [33], cetacean(s) [42], fox [38]
E	Cattle [24, 30, 38], sheep [15, 17, 27, 30], pig [3, 13, 15], alpaca [19], goat [17, 62], horse [56], yak [27], fox [38], deer [27], cat [27]
F	Cat [27, 51], cetacean(s) [42], pig [3, 33]
G	Rat [27, 63], mouse [63]
H	Grey seal [26], gull [26]

Unambiguous direct evidence that human giardiasis can be an example of a zoonosis, i.e. a human infection acquired from non-human hosts under “natural” conditions (via ingestion of *G. duodenalis* cysts excreted by animals), is limited. One study from the United Kingdom suggested that contact with farm animals (especially pigs) and with pets (especially dogs and cats) was a risk factor for giardiasis in human subjects [59]. Suggestive evidence that *G. duodenalis* can be transmitted between dogs and human subjects was obtained from a study in a tea-growing community in northeast India [55]. In this work, an association was found between the presence of *G. duodenalis* infection in human subjects and in dog(s) occupying the same household. For one such household, genetic identity between *G. duodenalis* in a dog and in human subject(s) was reported [55]. In this example, the direction of presumed inter-species transmission of *G. duodenalis* might have been either, or both, dog-to-human or human-to-dog. One caveat that applies to genetic studies of *G. duodenalis* that rely on faecal cysts as the starting material for molecular analysis is whether the presence of such cysts necessarily reflects infection, rather than resulting merely from coprophagy of faecal material containing cysts, and passage of these cysts through an animal’s gastrointestinal tract without causing infection [22].

Dogs have been infected with *G. duodenalis* of human origin, by oral administration of trophozoites or cysts of this organism [44]. There is an anecdotal report of an investigator developing giardiasis as a result of deliberately ingesting a gel capsule containing *Giardia* trophozoites that had originated from an animal host (a Gambian giant pouched rat) [31]. This work showed that animal-to-human transmission of *Giardia* infection can occur under experimental conditions. It is, however, unclear whether the result of the experiment just described constitutes evidence for the zoonotic transmissibility of *G. duodenalis*, under “normal field conditions”.

Genotyping of *G. duodenalis* organisms obtained from human subjects with *Giardia* infection has shown that assemblages A and B appear to be the only ones that undeniably cause human infections [15], although there have been occasional reports of the isolation, from human subjects, of *G. duodenalis* organisms that have genetic markers characteristic of non-A, non-B, assemblages [6, 49]. Mixed infections of human and non-human hosts with more than one assemblage of *G. duodenalis* concurrently have been described [15, 16, 18, 28]. Table 1 of the present article does not identify which infections, among those documented in the references cited, were part of a mixed infection resulting from more than one assemblage of *G. duodenalis*.

Individual *G. duodenalis* organisms can show sequence differences between different copies of the same gene (allelic sequence heterozygosity) [2, 7].

Although much of the literature on inter-species transmission of *G. duodenalis* has focussed on actual or presumed animal-to-human transmission, there is increasing evidence that *Giardia* cysts of human origin can contaminate the environment and infect wild mammals (which, in turn, may act as a reservoir for future infection of human subjects) [53]. The ability to identify *G. duodenalis* genetic assemblages has provided a level of precision and specificity that was lacking when essentially the only tool was morphological examination of trophozoites. For example, excretion of assemblage B cysts by Australian sea lions, and relative proximity of colonies of these animals to human settlements at coastal sites, speaks to the probability of initial infection of the animals by cysts of human origin [10]. Similarly, presence of assemblage A and B *G. duodenalis* infection in freely ranging gorillas may reflect human-to-gorilla transmission of the parasite [20, 21].

Of two outbreaks of human *G. duodenalis* infection in Canadian communities during the 1990s, one ceased and the other diminished after, in each case, a beaver excreting *G. duodenalis* cysts was removed from the water source supplying the respective community [40]. These observations suggested that the human cases of giardiasis had resulted from ingesting *G. duodenalis* cysts excreted by the beavers. The respective studies predated current knowledge of *G. duodenalis* assemblages. Archival material (*Giardia* organisms) from these outbreaks was, however, available for study by modern molecular techniques some two decades later [40]. Using such techniques, it was found that, in one of the outbreaks, the beaver was infected with assemblage A *G. duodenalis*, whereas the water contained *G. duodenalis* of assemblage B, and assemblages A and B were isolated from the infected human subjects. In the other outbreak, the beaver was found to be infected with assemblage B, whereas the infected human subjects included one with assemblage A infection [40]. Consequently, a straightforward causal relationship between the beavers and all the human cases was not found. Anthropocentric historical assumptions, that a relationship between *Giardia* infection in beavers and in human subjects merely involves beaver-to-human transmission of the parasite, have yielded to a more nuanced appreciation of environmental contamination by human-derived *G. duodenalis* cysts that may infect beavers [53].
